# Wi-Fi (2.4 GHz) affects anti-oxidant capacity, DNA repair genes expression and, apoptosis in pregnant mouse placenta

**DOI:** 10.22038/ijbms.2020.40184.9512

**Published:** 2020-06

**Authors:** Homeira Vafaei, Ghazal Kavari, Hamid Reza Izadi, Zahra Zare Dorahi, Mehdi Dianatpour, Afrooz Daneshparvar, Iman Jamhiri

**Affiliations:** 1 Maternal Fetal Medicine Research Center, Shiraz University of Medical Sciences, Shiraz, Iran; 2 Shiraz Neuroscience Research Center, Shiraz University of Medical Sciences, Shiraz, Iran; 3 Stem Cell Technology Research Center, Shiraz University of Medical Sciences, Shiraz, Iran; 4 Department of Human Genetics, School of Medicine, Shiraz University of Medical Sciences, Shiraz, Iran

**Keywords:** Anti-oxidant, Apoptosis, DNA repair, Placenta, Radiation exposure

## Abstract

**Objective(s):**

The placenta provides nutrients and oxygen to embryo and removes waste products from embryo’s blood. As far as we know, the effects of exposure to Wi-Fi (2.4 GHz) signals on placenta have not been evaluated. Hence, we examined the effect of prenatal exposure to Wi-Fi signals on anti-oxidant capacity, expressions of CDKNA1, and GADD45a as well as apoptosis in placenta and pregnancy outcome.

**Materials and Methods:**

Pregnant mice were exposed to Wi-Fi signal (2.4 GHz) for 2 and 4 hr. Placenta tissues were examined to measure the MDA and SOD levels. To measure SOD, CDKNA1, GADD45a, Bax, and Bcl-2 expressions were compared by real-time PCR analysis. TUNEL assay was used to assess apoptosis in placenta tissues. The results were analyzed by one-way analysis of variance (ANOVA) using Prism version 6.0 software.

**Results:**

MDA and SOD levels had significantly increased in exposed Wi-Fi signal groups (*P*-value< 0.05). Also, quantitative PCR experiment showed that SOD mRNA expression significantly increased in Wi-Fi signal groups. The data showed that CDKN1A and GADD45a genes were increased in Wi-Fi groups (*P*-value<0.05). The quantitative PCR and the TUNEL assay showed that apoptosis increased in Wi-Fi groups (*P*-value<0.05).

**Conclusion:**

Our results provide evidence that Wi-Fi signals increase lipid peroxidation, SOD activity (oxidative stres), apoptosis and CDKN1A and GADD45a overexpression in mice placenta tissue. However, further experimental studies are warranted to investigate other genes and aspects of pregnancy to determine the role of Wi-Fi radiation on fertility and pregnancy.

## Introduction

Reproduction is an important function of a living creature, which involves both female and male genital organs. Recently, evidence showed that both non-ionizing and ionizing radiations induce epigenetic changes, genomic instability and oxidative stress in reproductive tissues (1, 2). Radiofrequency (RF) radiation is part of the electromagnetic spectrum with frequencies ranging from 3 kHz to 300 GHz, which is below visible light and above extremely low-frequency fields (ELF-MFs) (3). Wireless fidelity (Wi-Fi) is a type of wireless local area network technology that acts in areas of the RF spectrum in the 2.45 and 5 GHz bands. Over these short distances, Wi-Fi devices only use low output power, usually limited to less than 2 W (4). 

Exposure to Wi-Fi signal might lead to absorption of some transmitted energy by cells. Moreover, concerns regarding exposure to radiofrequency electromagnetic field (RF-EMF) during perinatal life are elevated. Several reports showed that chronic and acute exposure to EMR can lead to morphological modification in tissues and biological cells (5, 6). It is suspected that oxidative stress and reactive oxygen species (ROS) generation might be the reasons for poor oocyte quality. Additionally, it was stated that oxidative stress might play a role in endometriosis-associated infertility and endometriosis development (7). Exposure to EMR in overproduction of ROS can damage cellular ingredients such as proteins, nucleic acids, and lipids. ROS can cause cellular injury by reducing antioxidants (enzymatic and non-enzymatic), triggering progressive dysfunction and eventually genotoxic events (8). Due to the aforementioned evidence, it was hypothesized that placental stresses during pregnancy can play a critical role in the pathogenesis of adverse pregnancy outcome. It was revealed that ionizing radiation induces DNA damage, which in turn causes cell damage and cell cycle arrest (9). 

Cyclin-dependent kinase interacting protein 1 (CDKNA1) and growth arrest and DNA damage inducible alpha (GADD45a) proteins play an important role in key cellular processes. The CDKNA1 and GADD45a proteins interact for the regulation of several cellular functions, including genotoxic stress, cell cycle control, senescence, and DNA damage repair. These proteins cause to facilitate cells to enforce cell cycle arrest in promoting DNA repair (critical for cellular responses to DNA breaks) (10, 11). 

To the best of our knowledge, the effects of exposure to Wi-Fi signals on placenta has not been assessed. Hence in the present study, we examined the effect of prenatal exposure to Wi-Fi signals on oxidative stress, apoptosis, and expressions of DNA repair genes including (CDK1A-GADD45a) in pregnancy outcome and placenta in a mice model.

## Materials and Methods

***Study design and population***

This study was designed as a prospective trial, conducted to examine the effects of prenatal (in uterus) exposure to Wi-Fi signals on the expressions of CDKNA1, and GADD45a in placenta and pregnancy outcome in an animal model. The institutional animal ethics committee of Shiraz University of Medical Sciences, which follows the National Institutes of Health guidelines for the care and use of animals, approved the protocols and experiments in this study. Pathogen-free 3-month-old plug-positive mice were enrolled in the study. Exclusion criteria included females with plug-negative after 4 days of mating and plug-positive females with unhealthy conditions during pregnancy.

We prepared pathogen free 3-month-old mice from the animal laboratory of Shiraz University of Medical Sciences. Male and female mice were mated over a 4-day period. Female mice were screened daily, and plug-positive females were separated from males. Females that were still negative after 4 days of mating were not included. Fifteen plug-positive females were assigned to 5 groups. Pregnant mice in case group were exposed to Wi-Fi signal-associated electromagnetic fields (2.4 GHz frequency band) by a D-Link Wi-Fi router (D-Link, DLink Corporation, Taiwan), starting 5 days after mating and ending 1 day before the expected delivery, and exposure protocol were grouped II-V (exposure protocols of 2 hr-30 cm, 2 hr-60 cm, 4 hr-30 cm, 4 hr-60 cm). During the exposure time, data was exchanged between a laptop and the modem at a distance of 5 meters (placed in another room). The Wi-Fi exposure source operated on power level of 1 W and the Specific Absorption Rate (SAR) at the distance of 30 cm in animals’ head level was 0.09 W/kg. The SAR comes from the following formula:

Specific Absorption Rate SAR=σ×E2md

Where σ, E and *m*_d _are the tissue conductivity, electric field and tissue density, respectively (12). Mice in the control group were exposed to the exact conditions as the other groups (without energizing the Wi-Fi router). When II-V groups were exposed to Wi-Fi, the control group was kept at a great distance in another room and we made sure that all the cellphone and Wi-Fi signals were discontinued, and the control group mice were kept in a special aluminum cage and made sure that there was no signal. We used an Aaronia portable spectrum analyzer to measure the electromagnetic quantities (Aaronia AG, Germany). After delivery, newborn mice were allowed to grow until 5 weeks of age, and then they were sacrificed via cervical dislocation. 

***Oxidative stress parameters***

*Sample preparation for measurement of antioxidant activity*

The placenta was rapidly isolated and washed by ice-cold isotonic saline and then stored at -80 °C. Afterward, placenta tissues were homogenized in a lysis buffer (Triton X-100, NaCl, Tris, EDTA, SDS, EGTA, HEPES, and PMSF) (Sigma, Germany) using a homogenizer (T10Bhomogenizer; Germany) and centrifuged for 45 min (13000 rpm at 4 °C). Then, the supernatant was used to measure antioxidant activity.

*Malondialdehyde assessment*

To measure malondialdehyde (MDA) level, 100 µl of supernatant, 5 µl of butilated toluene hydroxide, and 400 µl of %5 triclorostic acid were mixed, and the prepared solution was centrifuged for 10 min at 300 rpm. After removing 200 µl of the obtained solution, 150 µl of thiobarbitoric was added, and the mixture was placed in 95 ºC oven for 1 hr (All the materials used from Sigma, Germany). Then, it was preserved at -4 °C and the absorption rate was measured at 532 nanometer wavelengths (13). To determine the concentration of MDA, tetroxypropan was used as a control to plot the standard curve. The results are expressed as nanomoles of MDA per mg protein.

*Superoxide dismutase assessment*

Superoxide dismutase (SOD) activity was calculated by the procedure described by Madesh and Balasubramanian. It is a colorimetric assay involving the generation of superoxide by pyrogallol autooxidation and inhibition of superoxide-dependent reduction of the tetrazolium dye MTT [3-(4-5 dimethylthiazol 2-yl) 2,5-diphenyltetrazolium bromide] to its formazan, which is measured at 570 nm (All the materials used were from Sigma, Germany). The color evolved was stable for many hours and was expressed as SOD units (one unit of SOD activity was defined as the amount of enzyme causing 50% inhibition in the MTT reduction rate) (14). 

*Quantitative reverse transcription-PCR*

All placenta specimens were obtained through vacuum aspiration. Each specimen was flash-frozen in liquid nitrogen and used for quantitative reverse transcriptase polymerase chain reaction (qRT-PCR) analysis as described below.

SOD, CDKN1A, GADD45a, Bax and Bcl-2 genes expression were determined by qRT-PCR analysis using the β-actin gene as a reliable internal control. Total cellular RNA was isolated from tissue using RNA extraction kit (CinnaGen Inc., Tehran, Iran). The quantity and quality of the obtained RNA were checked by measuring the ratio of optical density (OD) of 260/280 nm using Nanodrop^TM^ spectrophotometer (Nanodrop; Thermo Fisher Scientific, Wilmington, DE, USA), and then stored at -80 °C until cDNA synthesis. The cDNA was then synthesized using 1000 ng of total RNA in a first-strand complementary DNA synthesis reaction by the help of RevertAid™ First Strand cDNA Synthesis kit (Thermo Fisher Scientific, Inc., Waltham, MA, USA).

Quantitative real-time PCR was performed using the ABI Biosystem step one and the RealQ Plus 2x Master Mix Green (Ampliqon Inc.,). In each reaction, 200 nM of each primer (Table 1) was added to target the specific sequence. Specific primers targeting β-actin, SOD, CDKNA1, GADD45a, Bax and Bcl-2 were designed, as shown in Table 1. The β-actin housekeeping gene was also used as internal control of qPCR reactions. The qPCR conditions were set for 10 min at 94 °C followed by 40 cycles of 15 sec at 94 °C, 60 sec at 58 °C and final extension of 7 min at 72 °C. The amplification signals of different samples were normalized to beta-actin Ct (cycle threshold), and then delta-delta CT (2^-∆∆CT^) method was used to compare mRNA levels of the test group versus control, which represents fold change in data analysis.

***TUNEL assays***

Three slides from each placenta were evaluated for cells apoptosis detection using TUNEL assay kit (TUNEL Assay, Roche Applied Science) according to the manufacturer’s instructions. Briefly, the placenta tissue slices were deparaffinised in xylenes and rehydrated at gradient concentration of ethanol. Proteinase k was added to increase membrane permeability. TUNEL MIX, and PI (Propidium iodide) were used for labeling and tracing. After washing, the slices were examined with a light microscope (Olympus BX40, Tokyo, Japan) at 400x magnification. The numbers of total and TUNEL-positive cells were counted in each field. The results were expressed as the apoptotic index (number of TUNEL-positive cell/total cell) ×100%.

***Statistical analysis***

All data in this study are analyzed by variance using the statistical program. All the data are presented as mean±SEM of at least three independent experiments and analyzed by one-way analysis of variance (ANOVA) (*post hoc: Tukey*) using GraphPad Prism software, version 6.0 (GraphPad Software Inc., San Diego, CA, USA) to compare control group. The *P-value* less than 0.05 was considered to be statistically significant.

## Results

**Figure 1:**
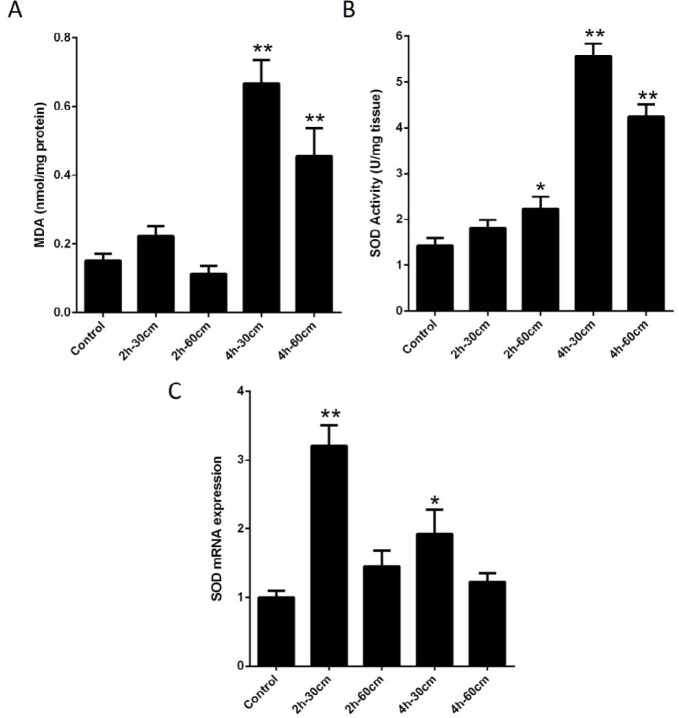
Effects of exposure to Wi-Fi radiation on antioxidant status in all study groups. A) MDA levels were evaluated in placenta tissues. B) Comparison of the SOD activity in placenta tissues (The activity was expressed as U/mg of protein). C) mRNA expression of SOD by qRT-PCR analysis. Bars represent mean ± SEM of 6 mice in each group. Statistical significance was tested using one-way ANOVA. * *P*-value <0.05; **, *P*-value<0.01. MDA: Malondialdehyde; SOD: Superoxide dismutase

The supernatant concentration of MDA had significantly increased in the groups IV and V (4 hr- 30 cm, 4 hr- 60 cm) compared to the control group (*P-value*<0.01). Two groups of II and III (2 hr- 30 cm and 2 hr- 60 cm) showed no significant difference with the control group (Figure 1-A). Also, the activity of total SOD was higher in the group IV (4 hr- 30 cm group) (5.56 U/mg tissue) compared to the control group (1.42 U/mg tissue) (*P-value*<0.01). Total SOD activity was also elevated in the group V (4 hr- 60 cm group) (4.24 U/mg tissue) versus the control group (*P-value*<0.01). However, the activity of SOD was higher in the group III (2hr-60cm) (2.23 U/mg tissue) as compared to the control group (*P-value*<0.05). In SOD activity, there were no significant differences between the group II (2 hr- 30 cm) and the control group (Figure 1-B). The expression analysis of SOD gene indicated that in placenta tissue exposed to Wi-Fi signals and high SOD mRNA expression levels, the expression pattern of SOD was significantly higher than the control tissues in groups II (3.20 fold) and IV (1.92 fold) compared to control group (*P-value*<0.01 and *P-value*=0.02, respectively) (Figure 1-C).

**Figure 2:**
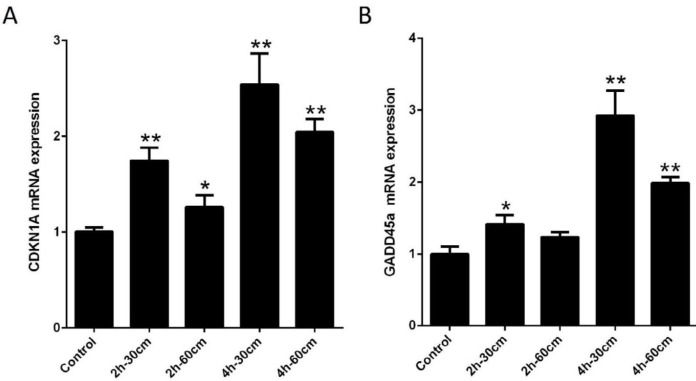
The fold change in genes expression of CDKN1A and GADD45a in all study groups. A) mRNA expression of CDKN1A. B) mRNA expression of GADD45a. Expression data relative to those of the reference gene from at least three independent assays are given as mean±SEM. Statistical significance was tested using the one-way ANOVA. * *P*-value< 0.05; **, *P*-value< 0.01. CDKNA1: Cyclin-dependent kinase-interacting protein 1, GADD45a: Growth arrest and DNA damage inducible alpha

The DNA repair genes (including CDK1A-GADD45a) in the placenta were also measured using the real-time quantitative PCR experiment. Overall, compared to the control group, there was an overall increase in the studied genes in the intervention groups. Real-time quantitative PCR showed that CDKN1A expression in all intervention groups was significantly different as compared to control group (*P-value*<0.05). The significance level of all groups (II-V) was less than 0.05 compared to the control group. In addition, the relative fold changes in GADD45a expression in the intervention groups were significantly higher than that of observed in the control group. Despite variations in different exposure groups, the maximum elevation for both genes was observed in mice exposed to Wi-Fi radiation in the 4 hr- 30 cm protocol. Figure 2 represents the fold change in DNA repair genes (CDK1A-GADD45a) expression of all the studied groups.

**Figure 3:**
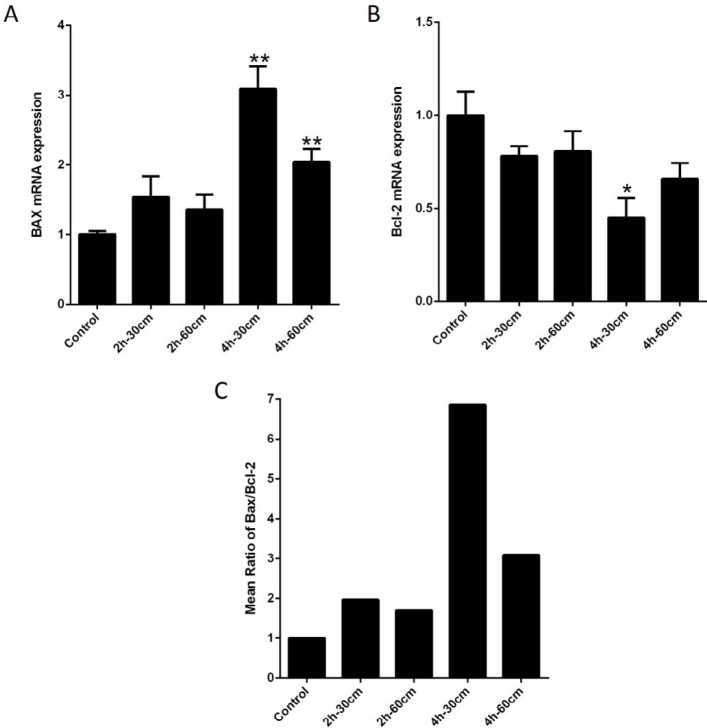
The effects of exposure to the 2.4 GHz Wi-Fi radiations on the pro-apoptotic genes (Bax), anti-apoptotic genes (Bcl-2) and Bax/Bcl-2 ratio of placenta. Bax and Bcl-2 gene expression levels were estimated by real-time quantitative PCR in placenta after the exposure to 2.4 GHz Wi-Fi radiations. A) Bax mRNA expression levels in placenta. B) The effect of 2.4 GHz Wi-Fi radiations on the Bcl-2 gene expression level. C) Ratios of Bax/Bcl-2 mRNA expression calculated from the mean value of each data. Expression data relative to those of the reference gene from at least three independent assays are given as mean±SEM. Statistical significance was tested using the one-way ANOVA. * *P*-value<0.05; ** *P*-value<0.01

**Figure 4:**
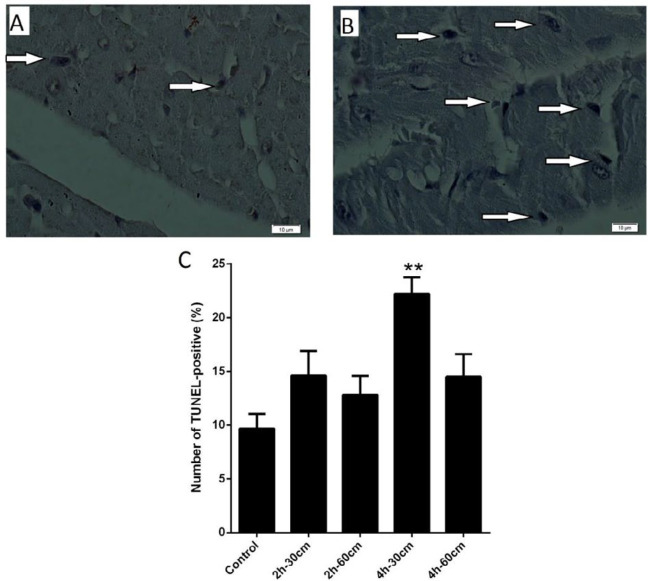
Representative TUNEL-positive nuclei in placenta after exposure to 2.4 GHz Wi-Fi radiations. A) The placenta tissue of control mice. B) The placenta tissue exposed to Wi-Fi signals (arrow indicating TUNEL-positive cells). Animals were exposed to 2.4 GHz Wi-Fi radiations, groups II-V (exposure protocols of 2 hr- 30 cm, 2 hr- 60 cm, 4 hr- 30 cm, 4 hr- 60 cm). C) Percentage of TUNEL-positive nuclei in different group. In group IV (4 hr- 30 cm), the apoptotic index had increased significantly. Data are expressed as mean±SEM. Statistical significance was tested using the one-way ANOVA. * *P*-value <0.05; ** *P*-value<0.01

We assumed that Wi-Fi radiation might induce apoptosis in the placenta by increasing the expression of Bax and reducing the expression of Bcl-2. The expression analysis results indicated that exposure to Wi-Fi radiation (group IV, 4 hr- 30 cm), significantly down-regulated the Bcl-2 (0.45 fold), compared to the control group (*P-value*<0.05). In accordance with real-time quantitative PCR results, the highest levels of Bax gene expression were found in group IV, 4 hr- 30 cm followed by group V, 4 hr- 60 cm (3.09 fold and 2.04-fold change**, **respectively**), **compared to the control group (*P-value*<0.05). There was no significant difference between the expression of Bax and bcl-2 in the 2 hr groups compared to the control group. Bax/Bcl-2 ratio is a measurable aspect of the apoptosis progression, which seems to determine the fate of apoptosis. The Bax/Bcl-2 ratio was increased in group V, 4 hr- 60 cm (6.86 fold). These data suggested that Wi-Fi radiation in the group V)4 hr- 60 cm) serves as an inducer of apoptosis through the activation of apoptotic genes as shown in Figure 3. To further explore apoptosis in placenta tissues, the rate of apoptosis was evaluated in experimental groups using TUNEL assay, shown in Figure 4. The percentage of apoptotic cells increased significantly in the group IV, 4 hr- 30 cm (22.2±1.5%) compared to the control group (9.66±1.3%) (*P-value* =0.0002). 

## Discussion

*In vitro* studies clarified the effects of RF radiation on chromosome aberrations, DNA damage, mutation, gene expression and cell transformation (15, 16). However, these studies focused on the cell lines for example breast cancer cells, immune cells, etc. (17). While few studies have examined neonatal or utero exposure to new signals such as Wi-Fi, many other studies established that exposure to very intense RF-EMFs might lead to deformities due to prolonged increase in organ temperature (18, 19).

Previous study showed the effect of exposure to Wi-Fi (2.4 GHz) on differentiation and proliferation of spermatogonia to be correlated with serum sex hormone levels. In parallel with a defect in spermatogenesis process, negative effects of Wi-Fi (2.4 GHz) on apoptosis status and histopathological changes of rat testis are possible (6). However, most studies that investigated teratology endpoints showed no deleterious effects by exposure to low (non-thermal) level EMFs (20, 21). Recent investigations reported that RF-electromagnetic wave (EMW) emitted from cell phones might lead to oxidative stress in human semen by keeping the cell phone in a trouser pocket in talking mode, which might harm spermatozoa by impairing male fertility (22). 

ROS are produced continuously by the placenta, and they are neutralized by antioxidants present in the tissue. Also, in order to study the role of oxidative stress in placental dysfunction, sensitive methods for the detection of oxidative stress are necessary. Oxidative stress is a critical factor in many problems during pregnancy; thus, uterine problems in pregnancy could result in an imbalance of antioxidant/oxidant activity when antioxidant capacity cannot keep pace with increased oxygen tension, leading to a chronic state of oxidative stress (23).

Previous experiments on the effects of extremely-low-frequency (ELF)-EMF on preimplantation embryos also hypothesized that the adverse effects of ELF-EMF on preimplantation embryos might be caused by the DNA damage in the embryos *in vitro* (24). However, as for the RF-EMF, few experiments were performed on its reproduction effects, especially on early stage pregnancy. The early stage of pregnancy is one of the most vital stages of reproduction, in which all tissues are sensitive to the toxic effects of the environment in comparison with the other steps during the life span (25). Consequently, the early stage of pregnancy placenta tissues was used in the present study to explore the possible effects of RF-EMF on reproduction.

Optimal fetal growth depends on functioning maternal, placental and fetal factors, the external environment, in combination with genetically predetermined growth potential. Fetal growth restriction (FGR) might occur as a result of defective function (26). Human placental development is recognized by trophoblast invasion into the uterine endometrium and its vasculature. The resulting changes will facilitate an increase in intervillous blood flow and hence, the exchange of molecules and nutrients between fetal and maternal blood. The transfer, as well as metabolic and endocrine functions of the placenta, reside primarily in the floating villi covered by the syncytiotrophoblast, a tissue that results from terminal differentiation of underlying villous cytotrophoblasts and their subsequent fusion. Anchoring villi establishes the physical connection of the placenta with the decidua predominantly by a subpopulation of cytotrophoblasts, known as EVT (extravillous cytotrophoblast). They are collected at the tips of the anchoring villi and form cell columns. Both villous and extravillous cytotrophoblast subpopulations arise by differentiation and proliferation from stem cells located within the cytotrophoblast layer of the chorionic villi (27). The trophoblastic cells in early stage of pregnancy are extremely sensitive to oxidative stress due to extensive cell divisions and the concomitant exposure of their DNA (28). CDKN1A, and GADD45a genes are, in essence, signal transducers that convert environmental and physiological stresses into various cellular stress responses including innate immunity, inflammation, and autoimmune diseases. Hence, both genes in placenta work as a hub to connect placental stresses (29, 30). Thereafter, we investigated the effects of Wi-Fi on the CDKNA1 and GADD45a expressions in placenta tissues. Both genes were more up-regulated with decrease in distance to Wi-Fi router and increase in time of exposure. Maximum genes expression was observed in mice exposed to Wi-Fi for 4 hr per day at distance of 30 cm from the router (7 days). CDKNA1 is one of the factors that promote cell cycle arrest in response to a variety of stimuli. The inhibitory effect of CDKNA1 on cell cycle progression correlates with its nuclear localization. CDKNA1 can be induced by both p53-dependent and p53-independent mechanisms. Some other important functions attributed to CDKNA1 include transcriptional regulation, and modulation or inhibition of apoptosis (31). On the other hand, GADD45a has been characterized as one of the vital players that contributes to cellular response to a variety of DNA damaging agents. Interestingly, the signaling machinery that regulates GADD45a induction by genotoxic stress involves both p53- independent and dependent pathways. Therefore, it can be assumed that overexpression of these genes promotes a variety of apoptosis and DNA damaging processes, by playing an important role in adverse pregnancy outcome (32). 

We showed that apoptosis marker was significantly higher than control after exposure to Wi-Fi signal, but to reveal the risk for animals, it requires more investigation and data to elucidate the mechanisms involved in Wi-Fi-induced apoptosis. On the other hand, investigating apoptotic damage is difficult to quantify *in vivo* for animals, because apoptotic cells are eliminated quite rapidly. The importance of these findings lies in the role of apoptosis, which is associated with problems in placenta and embryo (33). Hence, by limiting the Wi-Fi signal and consequently reducing apoptosis, there is a possibility for fewer risks.

Studies that investigated the impact of RF fields in the female genital system are scarce. To the best of our knowledge, there is no published report on the effects of RF on genes expression of placenta tissue. However, majority of studies focused on clinical pregnancy outcome or female and male infertility. A recent study showed that whole-body exposure to 2.14 GHz for 20 hr per day during gestation and lactation did not cause any adverse effects on pregnancy or the development of rats (20). The effects of lifetime exposure to UMTS-1966 MHz fields on reproduction and development were also investigated in Germany in four generations of mice (34). The WB SAR for adult animals was 0, 0.08, 0.4, and 1.3 W/kg, with a 24 hr per day exposure over lifetime. In this comprehensive study, no negative effect was found on pregnant females sacrificed on gestational day 18: the number of fetuses, normal or malformed, per litter. In addition, no negative effect was observed on the number or development of pups. In South Korea, Lee *et al*. exposed pregnant mice (WB SAR: 2.0 W/kg) to a CDMA (code division multiple access) signals or simultaneously to CDMA and WCDMA (wideband- CDMA) signals throughout the entire gestation period (35). The mice were exposed for 45 min twice per day, with a 15-min interval in between. On P18 of gestation, fetuses were examined for teratological parameters. Neither type of exposure caused any observable adverse effect on mouse fetuses. All the above experiments are in line with reviews that concluded that RF fields’ exposure had no effect on the gestation and development of rats or mice (18). Nevertheless, due to lack of genetic studies, it is difficult to determine the real role of RF in pregnancy. 

It should be noted that apoptosis status is induced by ROS through caspases-3 and -9 and cytochrome C, which in turn leads to a high rate of double and single DNA strand break (36). In the male reproductive system, the resulting imbalance in the redox status altered the sperm cycle progression and activated the apoptotic program through the rise of Bax, caspase 3, cytochrome C protein and gene expression and the reduction of Bcl-2 expression (37). In addition, near the ovulation time, an increase in several substances in the follicle can physiologically induce ROS and oxidative stress production. Follicles might be defenseless to oxidative stress induced by oocytes and becomes exposed to ROS continuously generated via the autooxidation of PUFAs (polyunsaturated fatty acids) of the follicles (38). 

The DNA repair proteins (including CDK1A-GADD45a) are increased following treatment with DNA-damaging agents and stressful growth arrest conditions. These proteins play a critical role in the regulation of DNA repair, cell growth, and apoptosis, in response to stressful conditions (39). The results of this study showed that Wi-Fi (2.4 GHz) (4 hr- 30 cm) might cause DNA damage, and as a result, the DNA repair genes increase significantly. This increase in genes expression revealed that Wi-Fi signals are more powerful with increasing timespan and reducing the distances, leading to more cell damage. Various studies showed the possible adverse biological effects of RF-EMFs on DNA, including an increased risk of cancer. A previous *in vitro *study reported that RF exposure at 1800 MHz induced DNA breaks in human lens epithelial cells (40). Furthermore, mitochondrial DNA defects and oxidative damage were detected in primary cultured neurons after 1800 MHz RF exposure. Activation of apoptosis is also considered to be involved in possible damage induced by RF-EMF. An *in vitro* study reported that radiation signals induced apoptosis in cells with the involvement of Bax and Bcl-2 (41). Our data showed that Wi-Fi (2.4 GHz) exposure increases apoptotic index and Bax/Bcl-2 ratio in the testes of exposed mice.

**Table 1:** Real-time PCR primers used in this study for the examination of gene expression

**Genes**	**Primer Sequences**	Sizes (bp)
CDKNA1	Forward: 5’- CAGAATAAAAGGTGCCACAGGC -3’	193
	Reverse: 5’- CGTCTCCGTGACGAAGTCAA -3’	
GADD45a	Forward: 5’- CTGCTGCTACTGGAGAACGAC -3’	152
	Reverse: 5’- CGACTTTCCCGGCAAAAACAAA -3’	
SOD	Forward: 5’- GCTGGCTTGGCTTCAATAAG-3’	90
	Reverse: 5’- GAATAAGGCCTGTTGTTCCTTG-3’	
Bax	Forward: 5’- AGCAAACTGGTGCTCAAGGC-3’	230
	Reverse: 5’- CCACAAAGATGGTCACTGTC-3’	
Bcl-2	Forward: 5’- GTGGTGGAGGAACTCTTCAG-3’	205
	Reverse: 5’- GTTCCACAAAGGCATCCCAG-3’	
β-actin	Forward: 5’- AGTGTGACGTTGACATCCGT -3’	120
	Reverse: 5’- TGCTAGGAGCCAGAGCAGTA-3’	

bp: base pair

## Conclusion

In summary, our results provide evidence that Wi-Fi signals increase oxidative stress in placenta tissue. It was suggested that CDKN1A and GADD45a overexpression in the placenta tissue was caused by exposure to Wi-Fi radiation, and increase in apoptosis-positive cells and Bax/Bcl-2 ratio in the placenta tissue of mice were observed, especially in 4 hr- 30 cm group. However, further experimental studies are warranted to investigate other genes as well as other aspects of pregnancy to determine the role of Wi-Fi radiation on fertility and pregnancy. 
